# Duodenal Gastrointestinal Stromal Tumor Causing Gastrointestinal Bleeding and Duodenal Obstruction in Third-Trimester Pregnancy Successfully Treated with Pancreaticoduodenectomy after Emergency Cesarean Section: A Case Report

**DOI:** 10.70352/scrj.cr.26-0237

**Published:** 2026-06-09

**Authors:** Ayumi Nakashima, Kazuki Takeishi, Kazuaki Hashimoto, Yoriko Nomura, Yusuke Kurokawa, Toyoma Kaku, Norifumi Harimoto

**Affiliations:** 1 Department of Hepatobiliary Pancreatic Surgery, National Hospital Organization Kyushu Medical Center, Fukuoka, Fukuoka, Japan; 2 Department of Gynecology, National Hospital Organization Kyushu Medical Center, Fukuoka, Fukuoka, Japan; 3 Department of Gastroenterology, National Hospital Organization Kyushu Medical Center, Fukuoka, Fukuoka, Japan

**Keywords:** gastrointestinal stromal tumor, duodenum, pregnancy, pancreaticoduodenectomy

## Abstract

**INTRODUCTION:**

We report a case of a pregnant woman suffering from gastrointestinal bleeding and duodenal obstruction caused by a gastrointestinal stromal tumor (GIST) that required pancreaticoduodenectomy after an emergent cesarean section.

**CASE PRESENTATION:**

A pregnant woman at 37 weeks of gestation presented with vomiting and melena and was found to have severe anemia. Imaging revealed an 8-cm tumor in the pancreatic head associated with duodenal stenosis. She underwent an emergency cesarean section, resulting in the delivery of a healthy infant. Subtotal stomach-preserving pancreatoduodenectomy was performed 7 days after delivery. Histopathological findings confirmed the tumor as a GIST. Her lactation support was provided by a lactation consultant starting on POD 1. The postoperative course was uneventful, and the patient was discharged on POD 10.

**CONCLUSIONS:**

This case highlights the importance of flexible treatment decision-making based on maternal condition and tumor-related complications, together with multidisciplinary perinatal support in patients requiring major surgery shortly after delivery to minimize the duration of mother–infant separation.

## Abbreviations

GIST
: gastrointestinal stromal tumor
SPN
: solid pseudopapillary neoplasm
EUS
: endoscopic ultra sonography

## INTRODUCTION

Gastrointestinal stromal tumor (GIST) are malignant mesenchymal tumors arising from immature mesenchymal cells located in the submucosal layer of the gastrointestinal tract.^1,2)^ GISTs predominantly occur in middle-aged and elderly individuals, and onset at a young age is rare.^1,3)^ We report a rare case of duodenal GIST presenting with gastrointestinal bleeding and duodenal obstruction in the third-trimester pregnancy, which was successfully treated by pancreaticoduodenectomy following an emergency cesarean section.

## CASE PRESENTATION

The patient was a 38-year-old woman who was noted to have an abdominal mass 2 and a half years before pregnancy, but declined further evaluation at her request. She subsequently became pregnant and was referred to our hospital for a pregnancy complicated by an abdominal tumor; at that time, she again declined further evaluation. At 37 weeks’ gestation, she presented to our emergency room with vomiting and melena. Severe anemia was observed, with a hemoglobin level of 5.3 g/dL. In addition, cardiotocography showed non-reassuring fetal status, which was thought to be secondary to maternal anemia. However, no improvement was observed even after transfusion of 6 units of red blood cells. Given the priority of fetal safety, further invasive diagnostic procedures such as upper gastrointestinal endoscopy were deferred. Therefore, an emergency cesarean section was performed, delivering a female infant weighing 2564 g. Further investigation revealed suggested duodenal obstruction and gastrointestinal bleeding due to the abdominal mass, leading to her referral. A fetal head-sized mass was palpable in the upper abdomen without tenderness. The laboratory data on admission and pre-pancreaticoduodenectomy day are summarized in **Table 1**. Blood urea nitrogen was elevated at 29 mg/dL, and severe anemia was present with Hb 5.3 g/dL on admission. Total protein was 5.7 g/dL and serum albumin was 2.7 g/dL. Coagulation tests were normal. Abdominal CT, performed on POD 3, revealed a cystic mass measuring approximately 8.5 cm in diameter in the pancreatic head, with dilated vascular development at its margins (**Fig. 1A**). There were no findings suggestive of infiltration into surrounding tissues, and no significant lymph node enlargement was noted. CT also demonstrated that the tumor was suspected to be causing compression from the descending to the horizontal portion of the duodenum, suggesting duodenal obstruction (**Fig. 1A**). In addition, the patient had persistent vomiting and was unable to tolerate oral intake. Based on these findings, duodenal obstruction was diagnosed. On POD 4, abdominal MRI revealed an approximately 8-cm tumor extending from the pancreatic body to the head. Its internal signal was hyperintense on T2-weighted images, suggesting a cystic tumor containing blood components/highly viscous contents (**Fig. 1B**). Upper gastrointestinal endoscopy was performed on POD 5; however, no obvious bleeding source was identified. Given that the patient was a young female and that the tumor was well demarcated with heterogeneous cystic components on imaging, a preoperative diagnosis of SPN was suspected. Endoscopic ultra sonography (EUS) was considered during the postoperative evaluation; however, because the patient had symptomatic tumor bleeding and duodenal obstruction requiring early definitive treatment, EUS-guided tissue acquisition was not performed, as it was unlikely to change the surgical strategy. On POD 5, a multidisciplinary discussion involving gastroenterologists, obstetricians, anesthesiologists and surgeons was held. Because the patient had symptomatic tumor bleeding and obstructive symptoms, prolonged delay was considered undesirable for both the mother and baby. Following the cesarean section, the patient received 6 units of blood transfusion, and her hemoglobin level improved from 5.3 to 7.2 g/dL. An additional transfusion of 4 units was administered on POD 6 prior to surgery, and her hemoglobin level reached 8.3 g/dL preoperatively. Despite suboptimal nutritional status, as reflected by hypoalbuminemia (albumin 2.7 g/dL) and low total protein (6.0 g/dL), her overall condition was considered adequate for surgery. At the same time, immediate pancreaticoduodenectomy just after cesarean section was considered highly invasive. Therefore, pancreaticoduodenectomy was performed on POD 7 after initial maternal stabilization and multidisciplinary evaluation. An emergency pancreaticoduodenectomy was performed 7 days after delivery. A midline upper abdominal incision was made for laparotomy. No peritoneal seeding, metastasis, or ascites was found in the abdominal cavity. Peritoneal lavage cytology was negative. The tumor was identified as an expansile mass in the pancreatic head without infiltration into surrounding organs. A pancreaticoduodenectomy with limited lymph node dissection (the first station only) was performed, and reconstruction was carried out using the modified Child method. The operative time was 4 h and 44 min, blood loss was 426 g, and no intraoperative blood transfusion was required. Oral intake of water commenced on POD 1. Her lactation support was provided by a lactation consultant starting on POD 1. Oral feeding resumed on POD 4. Biliary tube clamping commenced on POD 7. She received education regarding self-management of the external pancreatic duct tube, as well as dietary and nutritional guidance. The postoperative course was uneventful, and the patient was discharged home on POD 10 with the pancreatic duct tube left in place and kept on external drainage. The clinical course of this case is summarized in **Fig. 2**. Macroscopic findings of the resected specimen: A cystic tumor measuring 8.8 × 7.0 cm in diameter was identified, extending from the duodenal wall to the pancreatic head (**Fig. 3A**). The tumor occupied the lumen of the second portion of the duodenum and caused marked obstruction (**Fig. 3B**). The pathological findings of the resected specimen revealed a solid and cystic tumor with a fibrous capsule. Necrosis, hemorrhage, and cystic degeneration were observed. No extracapsular invasion was noted. The tumor consisted of spindle cells with mild nuclear atypia proliferating in an interdigitating pattern (**Fig. 3C**). It was DOG1-positive, c-kit-positive (**Fig. 3D**), and Ki67-positive at approximately 1%, leading to a diagnosis of duodenal GIST. According to the modified Fletcher classification,^4)^ the tumor was categorized as high risk because it was a duodenal GIST measuring 8 cm with 3 mitoses per 5 high-power fields. Adjuvant imatinib was not administered because the patient declined postoperative adjuvant therapy. At 9 months after surgery, the patient remains alive without recurrence, and the infant is doing well.

**Table 1:** Laboratory findings on admission and before pancreaticoduodenectomy (PD)

Biochemical findings			Hematological findings		
	Admission	Before PD		Admission	Before PD
Total protein (g/dL)	5.7	6.0	WBC (/μL)	17300	6200
Albumin (g/dL)	2.7	2.7	Hb (g/dL)	5.3	8.3
T-bil (mg/dL)	0.4	0.6	Hematocrit (%)	18.9	26.9
AST (U/L)	16	20	MCV	69.7	79.6
ALT (U/L)	7	18	PLT (10^4^/μL)	31.1	38.8
Na (mmol/L)	139	141	Coagulation findings		
K (mmol/L)	4.0	4.2	PT-% (%)	95	104
BUN (mg/dL)	29	12	PT-INR	1.03	0.97
CREA (mg/dL)	0.67	0.58	APTT (s)	23.9	24.7
Glucose (mg/dL)	106	78			
CRP (mg/dL)	0.03	0.57			

AST, aspartate aminotransferase; ALT, alanine aminotransferase; APTT, activated partial thromboplastin time; BUN, blood urea nitrogen; CRP, C-reactive protein; CREA, creatinine; Hb, hemoglobin; INR, international normalized ratio; K, potassium; MCV, mean corpuscular volume; Na, sodium; PT, prothrombin time; PLT, platelet count; T-bil, T-bilirubin; WBC, white blood cell count

**Fig. 1:**
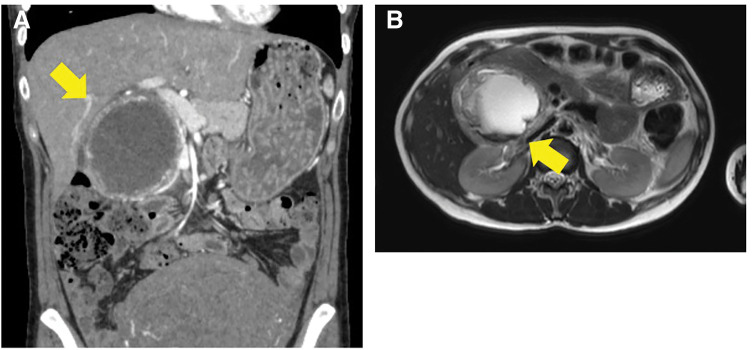
Preoperative images. (**A**) Contrast-enhanced CT shows an approximately 8.5-cm cystic mass in the pancreatic head with dilated peripheral vessels (arrows). The tumor compresses the descending and horizontal portions of the duodenum. (**B**) MRI demonstrates a heterogeneous mass with high signal intensity on T2-weighted images, suggesting a cystic tumor containing hemorrhagic components.

**Fig. 2:**
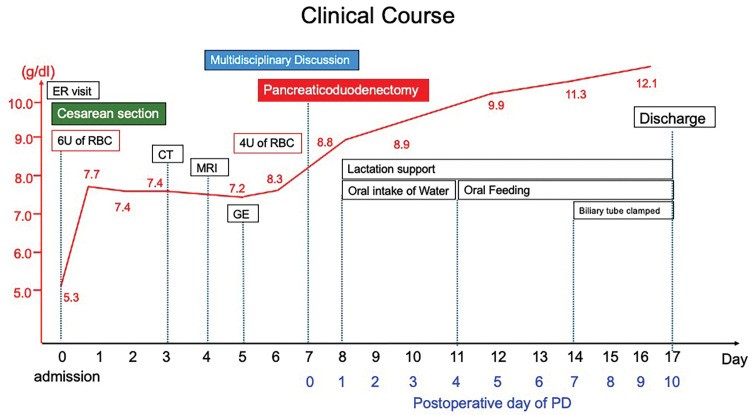
Clinical course and perioperative management. A schematic timeline of the clinical course from emergency admission to pancreaticoduodenectomy showing changes in hemoglobin levels. ER, emergency room; GE, gastrointestinal endoscopy; PD, pancreaticoduodenectomy; RBC, red blood cell transfusion

**Fig. 3:**
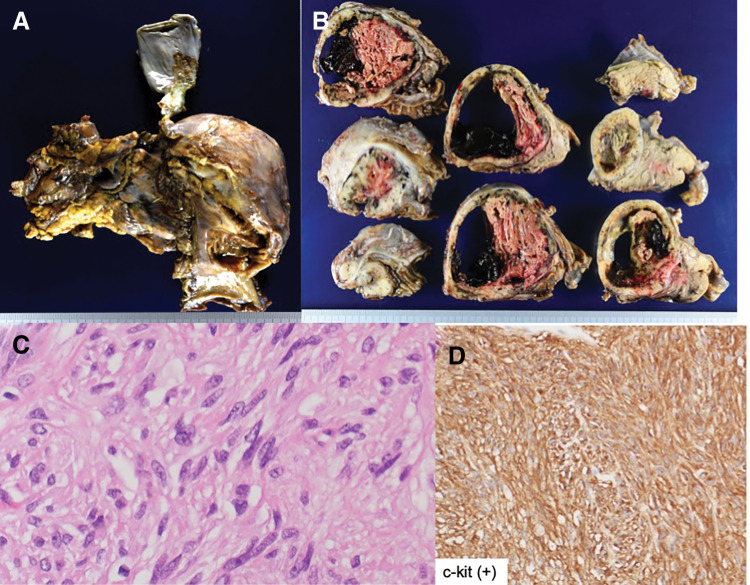
Macroscopic and pathological findings. (**A**) Gross appearance of the resected specimen. (**B**) Cut surface of the specimen showing a cystic tumor with intratumoral hemorrhage, extending from the duodenal wall toward the pancreatic head and occupying the lumen of the second portion of the duodenum. (**C**) Hematoxylin and eosin staining showing proliferation of spindle-shaped tumor cells with mild nuclear atypia arranged in an interlacing pattern. (**D**) Immunohistochemical staining showing positivity for c-kit (CD117).

## DISCUSSION

GIST is a relatively rare disease, accounting for 1%–2% of all gastrointestinal tumors and predominantly developing in individuals aged 60–65 years.^1,5)^ Given that GIST occurring at a younger age is uncommon,^3,5)^ its occurrence during pregnancy is extremely rare. To our knowledge, only 14 cases of GIST during pregnancy, including the present case, have been reported in the literature, and ours is the only case treated with pancreaticoduodenectomy.^6–18)^ Previously reported cases are summarized in **Table 2**.^6–18)^ Surgical resection is the primary treatment option for GIST,^1)^ but no clear guidelines exist specifying the appropriate timing for surgery in cases of GIST complicated by pregnancy. In the management of malignancy during pregnancy, maternal prognosis is generally prioritized, and necessary treatment, including surgery, should not be unnecessarily delayed solely due to pregnancy.^19,20)^ When determining the timing of surgery during pregnancy, gestational age is a key consideration. Nonurgent surgery is generally avoided in early pregnancy because of the risks of spontaneous abortion and fetal organogenesis. The second trimester is considered the safest period for surgical intervention, with relatively low risks of miscarriage and preterm delivery, and many reports have described favorable maternal and fetal outcomes when surgery is performed during this period. In third-trimester pregnancy, however, uterine enlargement may limit the operative field and increase the risks of preterm labor and perioperative complexity. Therefore, when the maternal condition is stable and tumor progression is slow, surgery may be deferred until after delivery once fetal maturity is achieved.^20–22)^ Particularly in third-trimester pregnancy, the decision to perform cesarean section and tumor surgery simultaneously or sequentially becomes a key consideration, necessitating multidisciplinary collaboration among obstetrics, surgery, and anesthesiology. An interval of several days after cesarean section allowed initial maternal hemodynamic stabilization and completion of diagnostic imaging, while avoiding an excessive delay in definitive treatment in the setting of ongoing bleeding risk and duodenal obstruction. Among the previously reported cases, 6 underwent surgery during the second trimester, whereas in the remaining 8 cases, including the present case, tumor resection was performed after cesarean delivery at 36 weeks or later. Treatment plans were individually determined based on gestational age, maternal condition, and tumor location/size. Early surgical intervention was chosen regardless of gestational age when complications such as tumor bleeding or intestinal obstruction were present. Conversely, if symptoms were mild, continuation of the pregnancy tended to be selected. In this case, the patient was transferred to our hospital during pregnancy due to pancreatic tumor complications. However, at her request, detailed evaluation was deferred, and the decision was made to continue the pregnancy and perform evaluation after delivery. During the third-trimester pregnancy, she developed severe anemia and intestinal obstruction. Judging the condition to be life-threatening for both mother and fetus, an emergency cesarean section was performed followed by a pancreaticoduodenectomy. Major surgery in the early post-cesarean period requires careful perioperative management. In this case, surgery was performed after initial maternal stabilization, including correction of anemia and assessment of general condition. Intraoperatively, efforts were made to minimize blood loss, maintain hemodynamic stability, and avoid unnecessary prolongation of the operative time to reduce surgical stress. Postoperatively, early mobilization and careful monitoring were emphasized, and attention was paid to minimizing mother–infant separation. In addition, this case was categorized as high risk according to the modified Fletcher classification. Therefore, postoperative adjuvant imatinib was considered. However, the patient wished to breastfeed and declined adjuvant therapy. Instead, close follow-up with imaging every 3 months was selected. At 9 months after surgery, no recurrence has been detected, and both the mother and infant are doing well. These factors resulted in favorable outcomes for both the mother and newborn. GIST during pregnancy is rare and can be challenging to diagnose. Flexible management, considering the condition of both mother and child through multidisciplinary collaboration, is crucial for determining the appropriate timing for surgery and selecting the treatment approach.

**Table 2:** Reported cases of GISTs during pregnancy

Author (year)	Age (year)	Tumor location	Surgical treatment timing (gestational week)	Fetal outcome	Maternal outcome
Valente et al.^6)^ (1996)	32	Stomach	Gastrectomy (28 weeks)	Alive	No recurrence
Lanzafame et al.^7)^ (2006)	29	Stomach	Gastrectomy (22 weeks)	Unknown	Unknown
Scherjon et al.^8)^ (2009)	25	Small intestine	Small intestine resection (15 weeks)	Alive	Unknown
Stubbs et al.^9)^ (2011)	31	Colon	Right hemicolectomy (36 weeks and postpartum)	Alive	Unknown
Coveney^10)^ (2011)	42	Left adnexa	Tumor resection (36 weeks and postpartum)	Alive	No recurrence
Igras et al.^11)^ (2012)	42	Duodenum	Duodenectomy (36 weeks and postpartum)	Alive	No recurrence
Gözükara et al.^12)^ (2012)	21	Omentum	Tumor resection (15 weeks, during pregnancy)	Unknown	Unknown
Goel et al.^13)^ (2013)	25	Abdomen	Non-surgical (imatinib during pregnancy)	Alive	No recurrence
Haloob et al.^14)^ (2013)	31	Small intestine	Small intestine resection (36 weeks and postpartum)	Alive	Unknown
Charif et al.^15)^ (2014)	42	Stomach	Exploratory laparotomy (36 weeks and postpartum); imatinib + gastrectomy	Alive	No recurrence
Charo et al.^16)^ (2018)	34	Small intestine	Small intestine resection (16 weeks)	Alive	No recurrence
Tanaka et al.^17)^ (2020)	29	Stomach	Gastrectomy (16 weeks, during pregnancy)	Alive	No recurrence
Chennouf et al.^18)^ (2022)	23	Stomach	Gastrectomy (37 weeks and postpartum)	Alive	Recurrence
**Our case**	**38**	**Duodenum**	**Pancreaticoduodenectomy (37 weeks and postpartum)**	**Alive**	**No recurrence**

GIST, gastrointestinal stromal tumor

When a mother undergoes major invasive surgery immediately after childbirth, mother–infant separation often becomes unavoidable, raising concerns about the psychological burden on the mother, the formation of the mother–infant bond, and the impact on the initiation of breastfeeding. Previous reports indicate that separation immediately after birth causes reduced maternal attachment behaviors, increased anxiety, and diminished maternal self-efficacy, with the potential for greater impact as separation prolongs.^23–25)^ In addition, delayed initiation of breastfeeding and reduced milk production are also recognized as significant challenges associated with separation. Therefore, studies indicate that these adverse effects of separation can be mitigated through early nursing and midwifery interventions.^25–27)^ Specifically, early postpartum pumping interventions are reported to not only maintain milk production but also foster the mother’s sense of active involvement in childcare, contributing to psychological stability and promoting the formation of the mother–infant relationship. In this case, the patient required radical surgery (pancreaticoduodenectomy) after cesarean section, necessitating temporary mother–infant separation. However, lactation support by a midwife began on POD 1, actively supporting both maintenance of milk production and maternal participation in childcare. This enabled the establishment of the mother–infant relationship in parallel with improvement in the mother's general condition. Furthermore, through active instruction on self-management of the extrapancreatic drainage tube, dietary guidance, and nutritional counseling, the patient was discharged smoothly on POD 10. In cases of malignant tumors complicating pregnancy that require highly invasive surgery immediately after delivery, it is considered crucial to minimize the impact of mother–infant separation by implementing a comprehensive perinatal support system. This system should incorporate not only perioperative surgical management but also midwifery and nursing interventions.

In this case, both the pancreatic duct tube and the biliary tube were externalized because the patient was in the early post-cesarean period, and every effort was made to minimize serious postoperative complications. Although firm evidence supporting external drainage is lacking,^28)^ we considered that it might facilitate postoperative management in this setting. The postoperative course was uneventful, with no pancreatic fistula or biliary leakage.

In the present case, the preoperative diagnosis was SPN, based on the patient’s young age, female sex, and the presence of heterogeneous cystic changes on imaging. However, because the patient developed gastrointestinal bleeding and bowel obstruction, multidisciplinary discussion led to the decision that emergency pancreatoduodenectomy was necessary. Furthermore, even in retrospect, pancreaticoduodenectomy would likely have been unavoidable because the main tumor was centered in the second portion of the duodenum and caused marked luminal obstruction, making limited resection and safe reconstruction technically difficult.

Regarding the extent of lymph node dissection, because the tumor was preoperatively diagnosed as SPN arising from the pancreatic head, pancreaticoduodenectomy with limited regional lymph node dissection was selected as the standard surgical approach, while standard lymphadenectomy equivalent to that for pancreatic ductal adenocarcinoma was considered unnecessary because SPN rarely involves lymph node metastasis.^29)^ In addition, given the emergency setting, limited regional lymph node dissection was selected. The final pathological diagnosis was GIST, for which routine extended lymphadenectomy is also generally not required.^30)^ Therefore, the extent of lymph node dissection performed in this case was considered appropriate in retrospect.

## CONCLUSIONS

A case of duodenal GIST during pregnancy, which caused acute bleeding and obstruction in the third-trimester pregnancy, was treated with a pancreaticoduodenectomy after prompt delivery, and we could save both the mother and the child safely. Individualized decision-making based on gestational age, maternal condition, and tumor behavior, together with multidisciplinary collaboration and perinatal support, is essential to achieve favorable outcomes for both mother and infant.

## DECLARATIONS

### Availability of data and materials

The data that support the findings of this study are available from the corresponding author upon reasonable request.
